# Function and Potentials of *M. tuberculosis* Epitopes

**DOI:** 10.3389/fimmu.2014.00107

**Published:** 2014-03-24

**Authors:** Juraj Ivanyi

**Affiliations:** ^1^Guy’s Hospital, Kings College London, London, UK

**Keywords:** tuberculosis, antigenic structure, epitope mapping, immunodominant epitopes, immunodiagnosis, immunotherapy, immunopathogenesis

## Abstract

Study of the function of epitopes of *Mycobacterium tuberculosis* antigens contributed significantly toward better understanding of the immunopathogenesis and to efforts for improving infection and disease control. Characterization of genetically permissively presented immunodominant epitopes has implications for the evolution of the host–parasite relationship, development of immunodiagnostic tests, and subunit prophylactic vaccines. Knowledge of the determinants of cross-sensitization, relevant to other pathogenic or environmental mycobacteria and to host constituents has advanced. Epitope-defined IFNγ assay kits became established for the specific detection of infection with tubercle bacilli both in humans and cattle. The CD4 T-cell epitope repertoire was found to be more narrow in patients with active disease than in latently infected subjects. However, differential diagnosis of active TB could not be made reliably merely on the basis of epitope recognition. The mechanisms by which HLA polymorphism can influence the development of multibacillary tuberculosis (TB) need further analysis of epitopes, recognized by Th2 helper cells for B-cell responses. Future vaccine development would benefit from better definition of protective epitopes and from improved construction and formulation of subunits with enhanced immunogenicity. Epitope-defined serology, due to its operational advantages is suitable for active case finding in selected high disease incidence populations, aiming for an early detection of infectious cases and hence for reducing the transmission of infection. The existing knowledge of HLA class I binding epitopes could be the basis for the construction of T-cell receptor-like ligands for immunotherapeutic application. Continued analysis of the functions of mycobacterial epitopes, recognized by T cells and antibodies, remains a fertile avenue in TB research.

## Introduction

Pathogenic bacteria produce a wide range of constituents, which determine their virulence and host responses following infection. In the case of *Mycobacterium tuberculosis* (Mtb), antigenic and immunomodulatory constituents may be considered as virulence factors, because they can act as “decoys,” triggering excessive immune responses which can lead to pathology of the lungs in active tuberculosis (TB), instead of host protection (1). Hence, immunological research has been essential for the study of pathogenesis as well as for the development of prophylactic vaccination and for the detection of latent infection. Detailed analysis of the specificity and of the phenotype of immune responses is mandatory in the desire to discover biomarkers for protective immunity, for predicting the risk of reactivation from latent infection and for developing immunotherapies, adjunct to chemotherapy.

Dissection of the antigenic structure of Mtb to its epitope constituents has been driven by the newly developed technologies, starting with hybridoma-produced monoclonal antibodies (2, 3), followed by recombinant DNA expression libraries (4), T-cell cloning and hybridomas (5), and DNA sequencing. More recently, new epitopes predicted within the whole Mtb genome on the basis of algorithms (“silico mapping”) (6) have a useful rate of empirical confirmation (7). The location of discontinuous and conformational epitopes recognized by antibodies can be predicted by integrated analysis of the dynamical and energetic properties of proteins (8). Mapping of T-cell epitopes within the known protein sequence used synthetic peptides with overlapping sequence (“pepscan”) and single-residue substitutions identified epitope cores, flanks, and key residues involved in binding to major histocompatibility complex (MHC) or T cell receptor (TcR) molecules.

Characterization of the membrane markers and cytokine profiles of responding T cells identified the existence of T-cell subsets and their regulatory networks. Emphasis on the T-cell phenotype and recently on the transcriptomic signature of T cells is currently expanding (9), but without full attention to antigen and epitope specificity. However, restoring the balance of knowledge between the functional phenotype and recognition specificities of T cells seems compelling.

The extensive knowledge on the mapping of antigenic epitopes has been cataloged and made accessible by the NIH IEDB database http://www.ncbi.nlm.nih.gov/pmc/articles/PMC2228276/; http://www.iedb.org/; http://help.iedb.org/entries/19150-user-documentation-iedb-version-2.

This inventory contains more than 1000 epitopes, mostly derived from only about 30 of the most immunogenic antigens, representing a very small fraction from the about 4000 known open reading frame proteins of the Mtb genome (10). Recently combined analysis of epitope predictions, high throughput ELISPOT, and T-cell libraries from latently Mtb-infected subjects, categorized the epitopes into prominent “antigenic islands” (11). The wider significance of epitope specificity of immune responses of Mtb-infected hosts has been reviewed recently (12).

This chapter points out the role of individual epitope specificities and aims to integrate this knowledge with different functions, relevant for the host–parasite relationship. Potentials for future research are targeted at improving the control of TB, particularly for vaccination, immunotherapy, detection of latent infection, and early diagnosis of infectious forms of active disease.

## MHC-Permissive Epitopes

Immunodominant epitopes were originally thought to be recognized in the context of only one or a few MHC class II alleles, though a number of genetically permissive epitopes were found in microbial pathogens. Initial mapping of CD4 T-cell stimulatory epitopes showed two hsp65 peptides recognized by several H-2-disparate mouse strains, one of them presented by both I-A and I-E molecules (13). Subsequent analysis of two glyco-lipoproteins and the α-crystallin (Acr) antigen identified the same few epitopes as immunodominant in a number of inbred strains of mice, carrying different H-2A alleles (14–16). H2I-A promiscuous recognition of p350–369 was demonstrated using CD4 T-cell hybridomas, though with allele-specific binding of IA polymorphic critical residues (17). Moreover, pepscan analysis revealed separate patterns of recognition by T hybridomas of the same H-2 haplotype, whereby every core residue was critical for at least one hybridoma, with only one substitution (74 Val → Ala) common to all hybridomas (18). While core residues were critical for both MHC and TcR binding, T-cell recognition was influenced also by the substitution of flanking residues (19).

The apparently abundant occurrence of MHC-permissive epitopes in tubercle bacilli may be an evolutionary consequence of selection of mutants carrying protective MHC-permissive epitopes. These organisms would have been advantageous to the pathogen, by being conducive to the longer survival of individuals, who were capable of aerosol transmission of the infection. This evolutionary concept is supported by the finding that T hybridomas from H-2Ab/d heterozygous mice had a higher frequency of IA-promiscuous recognition than hybridomas from each of the parental H-2 homozygous hybridomas (17). The IA-promiscuous hybridomas could also be stimulated with lower peptide concentrations, indicating TcR recognition of higher TcR affinity. Selection of MHC-permissive epitopes by low antigen concentrations in chronically Mtb-infected outbred populations would have had the advantage for the protection and survival of the infected hosts.

Analysis of HLA-DR heterozygous T-cell lines against the permissively recognized 91–110 epitope of the Acr antigen also showed superior stimulation in the context of heterozygous antigen presenting cells (APCs) (20). Moreover, stimulation in the context of DR-homozygous APCs showed that the HLA-DR haplotype influenced not only the magnitude, but also the IFNγ/IL-4 secretion profiles of the T-cell lines. The demography and evolution of Mtb lineages, virulence, and selection of epitopes of conserved structure were suggested also as an adaptation to other genetically diverse constituents in human macrophages, but without due consideration of the selective role of the HLA system for the selection of permissive epitopes (21).

The definition of MHC-permissive epitopes is mandatory for further development of both diagnostics and vaccines with a potential to function in large sections of genetically diverse human populations. HLA class II-permissive epitopes have been identified in a number of antigens of different structure, such as PstS1 glyco-lipoprotein (22), heat shock proteins hsp65 (23), and GroES (24), Acr (25), ESX proteins (26, 27), secreted proteins Ag85B (28) and MPB70 (29), and PE/PPE proteins (30) (Table 1). The abundance of human CD4 and CD8 T-cell responses to the respective epitopes was explained by their permissive binding to several HLA-DR (15, 22, 31, 32) and also HLA class I (26, 33, 34) molecules.

**Table 1:** **Mtb antigens with identified HLA-DR-permissive CD4+ T-cell stimulatory epitopes**.

Protein group	Antigen name	Gene accession no.	kDa	Epitope sequence	Reference
Glyco-lipoprotein	PstS1	Rv0934	38	1–20; 350–369	Jurcevic et al. (22)
Chaperonin stress proteins	α-Crystallin, Acr	Rv2031	16	91–110	Caccamo et al. (25)
	GroEL2, hsp65	Rv0440	65	61–75; 141–155	Mustafa et al. (23)
	GroES	Rv3418c	10	25–40	Chua-Intra et al. (24)
RD-1, ESX family	ESAT-6, EsxA	Rv3875	6	1–20	Tully et al. (27)
	CFP-10, EsxB	Rv3874	10	71–88	Shams et al. (26)
Secreted proteins	Ag85B mycolyl transferase	Rv1886c	30–32	91–108	Valle et al. (28)
	MPB70, mpt70	Rv2875	22	106–130, 166–190	Al-Attiyah et al. (29)
Cell surface	PPE family	Eight genes	3–316	Eight epitopes	Wang et al. (30)

Detailed analysis of the p350–369 epitope of PstS1 (35) showed a range of binding affinities to different DR molecules and identified the epitope core to be of 9–11 residues. Binding to both DR1 and DRB5*0101 shared F-354 as the common primary contact residue. Molecular modeling suggested that the peptide bound to DR1 in the elongated conformation as usual for MHC class II molecules, but in a “kink,” when bound to DRB5*0101, which is common for peptides bound to MHC class I complexes. The possible influence of different conformations imposed on the same peptide by distinct HLA alleles on T-cell responses has yet not been elucidated.

Substitution of single amino acids in the epitope core has been employed to identify both HLA-DR and TcR-binding contact residues within the DR17 restricted p3–13 epitope from the hsp65 antigen. Using this approach, TcR V gene families of human CD4 T-cell clones were analyzed in respect of the most immunodominant, HLA-DR promiscuous 91–110 epitope of Acr (25). The HLA-DR-binding and TcR-binding cores and contact residues were identified within 9-mer or 13-mer cores, which differed between the DR haplotypes. Notably, preferential TcR usage was demonstrated by the finding that the majority of clones used the BV2 TcR and contained a common R-L/V-G/S-Y/W-E/D sequence motif in the CDR3 region (36). These data may be useful to design peptides with altered HLA anchor residues or TcR interaction sites to increase their immunogenicity.

The “in silico” algorithms (ProPred1) predicted a number of HLA class I-permissive epitopes in histone or proteins of undefined function from Mtb (37). ProPred prediction of *Mycobacterium leprae* epitopes identified a number of both class I and II, HLA-permissive T-cell reactivity, in leprosy endemic populations in Brazil, Ethiopia, and Nepal (38). Comparing algorithms for HLA-binding promiscuity between ecologically diverse human microbial pathogens, found the promiscuity of Mtb epitopes to be of a similar degree as in HIV, *S. pyogenes*, or even higher for *B. anthracis* and *C. tetani* (39). However, these epitope predictions need empirical confirmation and should take into account that HLA binding affinities may not always associate with the magnitude of T-cell responses.

## Cross-Reactivity of Mycobacterial Epitopes

The antigens of Mtb are related to a number of proteins from non-tuberculous mycobacterial pathogens or commensal non-pathogenic mycobacterial species and more rarely with human proteins. These relationships are of interest, because environmental priming can influence resistance to Mtb, it can interfere or enhance the protective immune response to vaccination and may exclude from vaccine development, any molecules that can lead to autoimmunity. The latter category includes the chaperonins hsp65 and hsp71 with highly conserved sequences between prokaryotic and eukaryotic species and consequently extensive cross-reactivity between mycobacteria and humans (40). An hsp65 epitope at sequence 285–295, detected by mAb ML30 is strongly expressed on the surface of human cells with abundant mitochondria (40). Elevated expression was observed on monocyte-derived cells in different inflammatory diseases, including rheumatoid arthritis (41), atherosclerosis (42–44), and multiple sclerosis (45).

Analysis of antigen homologs from different species of mycobacteria showed that cross-recognition by T cells requires sharing fewer amino acids than cross-reaction by antibodies. Thus, polyclonal and monoclonal antibodies to ESAT-6 from Mtb and *M. leprae*, which share only 36% amino acids, are all strictly species-specific (46). In contrast, recombinant ESAT-6 from both species are similarly recognized by T cells from individuals, who were exposed to either tuberculous or leprosy infection (46). Cross-reactivity at the level of T-cell, but not B-cell recognition can lead to the recall of antibody production with specificity for the initial antigen (termed: “original antigenic sin”) (47). This interpretation was given to the finding of elevated antibody levels against Mtb-specific epitopes in lepromatous leprosy patients from TB endemic areas (48). However, *M. leprae*-specific antibodies in patients with active TB were not raised, since these patients were probably not exposed to *M. leprae* infection.

Cross-reactivity without sequence homology, i.e., mimicry, has been observed with a broad range of molecules of different structure, such as lactoferrin, transferrin, and proteoglycan. Another example of mimicry is the cross-recognition by CD4 T cells of an octamer epitope on two unrelated mycobacterial proteins, which is immunodominant for the 19-kDa protein of Mtb and cryptic for the 28-kDa protein of *M. leprae* (49, 50). Assumptions that epitope-based mimicry between proteins could lead to unsuspected cross-sensitization, maintain T-cell memory, or lead to autoimmunity, need further study.

Despite wide sequence homologies, heat shock proteins contain also species-specific epitopes. Increased Mtb-specific antibody levels were reported for hsp65, hsp71 (51, 52) in patients with active TB, including patients with smear-negative disease, which remains a diagnostic obstacle. Elevated serum antibodies to mycobacterial, but not to human hsp65 in Crohn’s disease, implied a pathogenic role of mycobacteria, whereas antibodies in ulcerative colitis bound to human hsp65 (53). Further support for the mycobacterial pathogenesis of Crohn’s disease came from the finding of elevated antibody levels against three different antigens derived from *Mycobacterium paratuberculosis* (54).

Immunodominant species-specific T- and B-cell epitopes can be found in a mycobacterial 10-kDa GroEL heat shock protein despite its highly conserved amino acid sequence. Despite a 90% sequence identity with Mtb, studies in mice identified two *M. leprae*-specific closely overlapping CD4 T-cell epitope cores (24–34 and 28–34), restricted by H-2Ad and H-2Ed, respectively and overlapping with an *M. leprae*-specific mouse mAb (ML6 and 10) epitope at residues 25–31 (55). The lack of antibody response to this epitope in lepromatous leprosy patients was suggested to be due to the T-cell epitope overlap.

The CD4 T-cell epitope repertoire of GroES was investigated also in TB and leprosy patients. The N-terminal (1–16) peptide (residues 1–16) was specifically stimulatory in the majority of active TB patients (56), while none of the other peptides was discriminatory. On the other hand, peptide 25–40 (29–37 core) of *M. leprae*, but not of Mtb sequence was specifically stimulatory in tuberculoid leprosy patients; this peptide bound to a number of HLA-DR molecules, of which HLA-DRB5*0101 had the strongest affinity (24). Four other leprosy-specific epitopes were identified on the 35-kDa protein of *M. leprae*, which has a homologous constituent in *M. avium*, but not in Mtb (57). Analysis of epitope specificities explained the phenomenon of split leprosin/tuberculin anergy of skin hypersensitivity in a proportion of leprosy patients. Thus, blood T-cell proliferative responses were found to be diminished to cross-reactive antigens, but elevated toward the predominantly Mtb-expressed PstS1 antigen and the Acr epitope 71–91 (58).

A special case of cross-reactivity is the induction of epitope-specific immune responses by anti-idiotype (Id) antibodies, acting as the epitope’s “internal image.” This was demonstrated for both mouse and human CD4 T-cell responses, using rabbit anti-Ids raised against PstS1-specific mouse mAbs (59, 60). However, the corresponding structural determinants remained undefined and the approach has been overtaken by the more exact recombinant DNA and synthetic peptide technologies. Id specificities have also been identified on anti-DNA autoantibodies stimulated probably by bacterial polyclonal B-cell activation in patients with TB and leprosy (61, 62).

## T-Cell Epitope Analysis in Latent Mtb Infection

*Mycobacterium tuberculosis* infection is routinely being monitored by skin delayed type hypersensitivity (DTH) reactions against tuberculin (PPD), a crude extract from *M. tuberculosis*. This test has poor specificity, due to cross-reaction with environmental mycobacteria and vaccination by Bacillus Calmette-Guerrin (BCG). The discovery of Mtb-specific, immunodominant, HLA-permissive epitopes, led to the use of synthetic peptide for the *in vitro* stimulation of blood T-cell responses. The numerous candidate peptides described (not reviewed here) have all been selected from several antigens on the grounds of better specificity than PPD, but they are performing at lower sensitivity than PPD. Improvements to sensitivity by using peptide pools has however had a limited impact, because of overlapping, rather than complementary recognition by the T-cell repertoire (22). The increase in stimulation by a pool of eight different peptides over the best single peptide (p91–110 from Acr) has been merely marginal. This outcome was attributed to HLA permissiveness and to competition between peptides for a limited number of binding sites on the HLA class II molecules of the APCs. This latter explanation has been supported by the finding of a declining response trend to higher concentrations of pools, but not of single peptides.

Nevertheless, commercially available IFNγ detection kits (IGRA), e.g., QuantiFERON-TB Gold (QTF-G), T-SPOT.TB etc. have been widely used for the specific detection of latent Mtb detection. They usually contain a mixture of several epitopes, predicted by algorithms of the Mtb-specific ESAT-6 and CFP-10 RD-1 antigens (absent from BCG and environmental mycobacteria). However, the use of these kits in areas highly endemic for TB is not of great added value for diagnosis. Recent side-by-side analysis of constituent antigens indicated potentials for further improvement of the test kits (63). Peptide cocktails have also been useful for the diagnosis of bovine TB in cattle, using either skin test or blood assays (64, 65). The blood INFγ assay (BOVIGRAM) readout has been further enhanced by adsorbing the peptides onto a range of microparticulate and nanoparticulate substrates (66). Detection IFNγ-induced protein (IP-10), which is produced in 100-fold greater amounts than IFNγ, has been developed for a simplified and more robust lateral flow test than IGRA (67). Notably, satisfactory results were obtained using dried plasma spots, amenable for conventional postal transport (68).

In view of the possibility that sequence variations in epitopic regions between clinical Mtb isolates might affect the results of IFNγ assays, human clinical samples were sequenced to identify substitutions that may have an impact on immunogenicity. A number of sequence polymorphisms (SNPs) have been revealed in the epitope regions of EsxB and EsxH genes (69), with evidence for recombination events, which may truncate the corresponding protein. Even single-residue differences altered the responder frequencies to these antigens from *M. bovis* isolates (70). Hence, immune variation may influence the diagnostic performance of kits, which contain epitopes from the ESX proteins.

## T-Cell Epitope Repertoire in Active TB

Commercially available IGRA assays routinely used in clinical practice do not distinguish reliably between active TB and latent infection and have limited value for predicting the risk of developing active TB (71). Therefore, it has been of interest to search, if fine analysis of epitope specificities could improve the diagnosis. Proliferation assays of blood T cells from patients with active TB recognize a smaller number of Acr epitopes than sensitized healthy subjects (Table 2) (32). Similarly, patients with leprosy recognize fewer GroEL epitopes than healthy contacts (24). These findings corroborate with the previously known skin DTH anergy to PPD in a fraction of active TB patients and with the development of leprosin anergy in multibacillary leprosy. The search for epitopes, which would distinguish patients from latent infection, yielded a promising result only for the amino-terminal peptide of GroES (1–16) (56). The selective power of this peptide is surprising, considering its overlap, except for one residue, with the *M. leprae* sequence and is in need of confirmation with more clinical samples.

**Table 2:** **Recognition of fewer epitopes in active disease than in latently infected subjects**.

Subjects (no. tested)	Peptides from	Frequency (%) of responders^a^, no. of test peptides (p)		
From London^b^	Acr	1–3 p	4–6 p	>6 p
Healthy PPD+ skin test (25)		24	52	24
Active TB (38)		52	30	18
From Bangkok^c^	GroES	1–4 p	5–10 p	>10 p
Healthy family contacts (12)		<1	58	42
Tuberculoid leprosy (18)		33	56	11

^a^Blood mononuclear cells of responders had at least threefold elevated ^3^H-thymidine uptake in cultures containing the test peptide over medium alone.Data from:

^b^Friscia et al. (32);

*^c^Chua-Intra et al. (24)*.

A reciprocal approach to the differential diagnosis has come from the finding of selective T-cell anergy in active TB in respect of the carboxy-terminal epitope p350–369 of the PstS1 antigen, which is strongly immunogenic in latently infected subjects (31). The lack of blood T-cell response can be due to sequestration to the site of disease (e.g., pleural fluid) and the ratio of T cells between these compartments is influenced by chemotherapy (72, 73). The post-chemotherapy recovery of blood response seemed more pronounced for T cells reacting with the Acr peptides, than those responding to the PstS1 peptides, which could be explained by differences in expression between replicating and chemotherapy-generated persister organisms. While dissecting of active from latent TB T-cell repertoire merely on the grounds of epitope specificity had failed to reach consensus (72), significant differences in their cytokine secretion are represented by elevated number of polyfunctional T cells (secreting IL-2, IFNγ, and TNFα) in active TB (74).

HIV-infected subjects have a high risk of reactivating their latent Mtb infection. Predicting this outcome better than just on the grounds of declining CD4 counts, would be of prime interest. The ratio of IFNγ ELISPOT counts in response to RD-1 peptides over CD4+ T-cell counts, greater than 0.21, showed 100% sensitivity and 80% specificity for active TB (75). However, the finding of 19% non-responder TB patients limited the diagnostic scope of this study. PPD-stimulated T cells carry higher levels of HIV DNA and depleted sooner than T cells of other specificity (76). This could explain the frequent reactivation of latent Mtb infection in HIV+ subjects. The antigen and epitope specificities involved have not been studied much beyond the crude PPD extract and the mechanism, which renders Mtb-reactive cells more permissive to HIV infection, is not understood. One possibility could be the lower stimulatory dose of the HLA-permissive epitopes for T cells with high affinity TcRs. This mechanism would imply selective recognition of certain epitopes, which would be rewarding to identify in the future. So far however, attention has been directed toward the study of the CD4 T-cell phenotype, characterized as CXCR3+ CCR4+ CCR6+ CD57− IFNg+ IL-17+ IL-2^hi^ MIP1b^low^ for the highly HIV-permissive PPD-reactive and CXCR3+ CCR4− CCR6− CD57+ IFNg+ IL-2^low^ MIP1b^high^ for low HIV-permissive CMV and other virus-reactive T cells (76).

## Association of HLA-DR and Antibody Epitope Specificity with TB

Susceptibility to TB is considered to be under the influence of multiple genetic loci, including HLA alleles. HLA-DR2 was found inherited more frequently in offspring with pulmonary TB, from both diseased and healthy parents (77) and associated with sputum-positive, but not with sputum-negative active pulmonary TB (78). DR2 alleles in sputum-positive TB associated also with elevated antibody levels to two epitopes of the PstS1 lipoglycoprotein antigen (79), but not with the similarly elevated antibody levels to epitopes of three other antigens of diverse nature (Acr, 19-kDa lipoglycoprotein, and lipoarabinomannan). The intriguing aspect of this finding is that both the genetic and immunological specificities were identified.

To explain the DR2 gene control of TB susceptibility, it has been proposed (80) that T-cell recognition of DR2-restricted PstS1 epitopes may lead to a Th2 response, producing IL-4 and IL-10 cytokines, which can lead to the development of lung pathology, rather than host protection. This hypothesis is supported by the finding, that selection of epitopes presented by B cells, rather than dendritic cells, diverted T cells from protection toward pathogenicity in *Leishmania* infection (81). Thus, the specificity of antibody responses during active TB could guide toward antigens, containing potentially pathogenic Th2 recognized epitopes. The search for such T-cell epitopes on the PstS1 antigen so far did not yield supportive data. Epitope specificity was determined only for Th1 cell clones which were mostly HLA-DR promiscuous (82) with all immunodominant epitopes of PstS1 binding to several HLA-DR molecules (22). However, these assays may not be suitable to reveal a DR2-restricted presentation of the same epitope to Th2 T cells. Development of assays for the mapping of Th2 cell stimulatory epitopes will be important to explain the mechanism of HLA class II-mediated influences on the development of multibacillary TB.

Analysis of some of the above raised aspects had been approached in mouse experimental models. Notably, influence of H-2 genes (Db or lack of I-E expression) was observed on the late progression of intraperitoneally delivered infection and pathology in the lungs, when spleen and liver bacillary counts remained stationary (83). Though this model of selective multibacillary lung disease seems relevant, the antigen specificity of the underlying immune responses was not identified. Although antibody responses to different antigens and epitopes is under H-2A control following immunization with soluble antigens in adjuvants (84, 85), there is no clear corresponding evidence following Mtb infection. On the other hand, antibody responses to hsp65 and hsp71 antigens (86) and liver granuloma formation (87) following Mtb infection were associated with non-H-2 genes.

Further analysis of the Th2 (T-helpers for B-cell responses) epitope repertoire also needs further studies in mouse models, addressing the topographical relationship between CD4 T-cell and B-cell stimulatory epitopes (88). Though using merely proliferation assays, it appeared, that PstS1 antigen immunized mice produced CD4 T cells, but not antibodies against the p65–83 peptide, which contains a non-overlapping cryptic B and an immunodominant T epitope core, while T-cell help was “delegated” probably to distantly located linear or conformational B-cell epitopes (Table 3) (89). A functional association between topographically distinct epitopes was suggested also by the finding that a single amino acid mutation of epitope core of the 19-kDa antigen abrogated T-cell, but not the B-cell immunogenicity (90).

**Table 3:** **Differences in epitope recognition between immunized and infected C57Bl/10 mice**.

Foot pad injection	T-cell proliferation to peptides				Antibody level		
	44–64	65–83	123–143	350–368	p1–20	p201–220	TB71
Recombinant PstS1^a^	−	++	++	−	−	+++	++
Heat-killed H37Rv^a^	−	−	+	+	+++	−	+
H37Rv infection	+	+	−	−	−	−	++

*Magnitude of the immune response: stimulation indices: −, <1; +, 1–10; ++, 10–20 of spleen and lymph node cells 7 days after immunization*.

*ELISA peptide binding or TB71 mAb competition titers: –, <10; +, 10–100; ++, 100–1000; +++, >1000 in sera harvested 12 weeks after first inoculation*.

*^a^Antigen in incomplete Freund’s adjuvant followed by three boosters without adjuvant. Data from Vordermeier et al. (89)*.

## Peptide Epitope-Based Vaccination Against TB

Most research toward a better vaccine against TB has been based on boosting immunity after BCG priming. The choice of antigen for this purpose has been to some extent subjective, though usually targeting proteins which appeared as most immunogenic in Mtb-infected individuals or experimental animals. Further breakdown of the immune repertoire to individual epitope specificities showed that both immune recognition was influenced by the nature of the immunogen. Thus, CD4 T cells recognized different peptides, when mice were vaccinated with either PstS1 antigen or heat-killed Mtb or infected with H37Rv bacilli (Table 3) (89). Antibodies reacted to different epitopes following vaccination, but bound only to conformational epitopes following infection. CD8 T cells also recognized different peptides following vaccination or Mtb infection (91, 92). These results indicate that the nature of the immunogen could influence antigen processing, which may deviate T-cell help from one to another B-cell epitope. Substantial differences in protection, cytokine profile, and recognition of T-cell epitopes of Ag85A or the Ag85B–ESAT-6 fusion protein were observed after its presentation either expressed in adenovirus vector or with an adjuvant (93). Disparities were observed also in response to Ag85A and its immunogenic peptides, when inoculated intranasally or parenterally (94).

DNA gun bombardment has been used for the mapping of T-cell epitopes and the effect of self-adjuvanting domains. Several CD4+ and one CD8+ T-cell epitopes were identified on the DNA-binding protein 1 (MDP1) antigen (95), while spleen CD4 T cells from HLA-DRB1*0401 transgenic mice recognized only the p191–210 epitope on the MPT51 antigen (96). A fusion DNA vaccine incorporating the HSP70 C-terminal domain (as adjuvant) and MPT51 (as target antigen) stimulated CD4, but not the CD8 T-cell response (97). Though documenting immunogenicity, these results need to be extended for protection against challenge.

Vaccine design could benefit from modifying the structure of peptides to increase their immunogenicity, while conjugation of a MHC-permissive peptide could abrogate genetic restriction for another MHC-restricted epitope. Studies in this direction showed that orientation between two epitopes within a synthetic peptide dimer can profoundly influence immunogenicity (98). Orientation of peptides played a role also for chimeric peptides constructed by recombinant DNA technology (99). Immunogenicity can be increased also by extension of an epitope core with non-native flanking residues (100) or by covalent attachment to biodegradable amphoteric branched chain polypeptides (101). Lipoylation of the MHC-promiscuous 91–110 peptide of Acr inoculated without any adjuvants was reported to enhance the immunogenicity and imparted protection against aerosol Mtb challenge in both mice and guinea pigs to an even better extent than BCG (102).

## Epitope-Specific Serodiagnosis

*Mycobacterium tuberculosis* species-specific mAbs had been used in a competition serodiagnostic test for TB and leprosy, preceding the purification of target antigens (103, 104). The mAb competition test has the advantage of higher sensitivity due to its low background values, which allowed the use of 20 times lower serum dilutions (i.e., 1/5) than standard ELISA tests (1/100). The competition assay also discriminated species-specific from cross-reactive epitopes on a number of antigens and identified several associations between antibody specificity and clinical aspects of TB (105, 106) (see also chapter by G. Bothamley). Epitope-specific antibody levels can be representative for both specificity and sensitivity of the whole antigen (e.g., PstS1) (51). However, the mAb competition test was more specific than binding to the whole lipoarabinomann, by targeting a Mtb-specific epitope or avoiding detection of contaminants. Epitope-specific titers also reflected the clinical form of TB, when related to titers against the whole 19-kDa lipoprotein (107).

Several serological surveys showed that serum antibody levels are consistently elevated in the great majority of sputum-positive TB, but not in sputum-negative disease (108). Though the latter aspect is greatly limiting the diagnostic application of serology, this hindrance was not acknowledged during the uncontrolled marketing of commercial kits. On the other hand, detection of antibodies in the cerebrospinal fluid is of particular value for the diagnosis of TB meningitis (109), where rapid detection can be life saving. Due to the high sensitivity, low cost, and operational advantages, serological screening has been suggested for active case finding in high-risk populations for multibacillary infectious patients (108, 110). Their early diagnosis could reduce the transmission of Mtb infection and therefore represents an important epidemiological, rather than clinical objective. A similar rationale could apply to leprosy, where antibody levels are elevated in the infectious multibacillary lepromatous, rather than the paucibacillary tuberculoid form (3).

A recent advance in epitope screening has been the high-content peptide microarray chip technology, involving the testing of thousands of different peptides. This approach showed that several epitopes are differentially recognized by IgG antibodies in pulmonary TB sera (111) and identified epitope “hotspots” within a number of protein antigens with similar patterns for patients of different genetic background. These linear epitopes are likely to detect a different repertoire than serology based on whole protein molecules, which detects mostly antibodies against conformational epitopes. Further clinical evaluation, particularly comparing multi- and paucibacillary forms of active TB, seems warranted.

## TcR-Like Ligands

Tubercle bacilli multiply and persist predominantly in macrophages, which display on their surface HLA-bound antigenic mycobacterial peptides, which are recognized by T-cell receptors (TcR). T cells can impart protection, but their excessive reactions can lead also to inflammatory pathology, characteristic of active TB. To avoid the latter outcome, suitable T-cell receptor-like constructs could be protective, without the accompanying undesirable T-cell-mediated inflammation. Immunotherapy using soluble TcR ligands could kill macrophages infected with both replicating and dormant Mtb organisms. Based on this hypothesis, TcR-based immunotherapy could be used as an adjunct to chemotherapy and be particularly useful to HIV-infected TB patients, many of whom being immunocompromised, cannot be protected by active vaccination. Therefore, TcR immunotherapy might be better than “therapeutic” active vaccination and also more efficient than passive antibody therapy, which can target probably only extracellular Mtb bacilli (112, 113). Unlike antibodies to B-cell epitopes [including those directed against overlapping T and B epitopes (114)], TcR-like ligands would be directed against epitopes, displayed in complex with MHC molecules on the surface of Mtb-infected cells.

Development of immunotherapeutic agents with TcR specificity has become feasible with the introduction of two technologies: (1) single chain antibody fragments (scFv) with TcR specificity can be selected from phage antibody libraries (115–118); (2) monomeric high affinity soluble human TcRs (mTcRs) have been expressed from cloned CD8 T cells and produced within the cytoplasm of trxB gor mutant *E. coli* strains (119). TcR-like mAbs against HLA class I presented epitopes of tumors (120) and virus-infected cells are being developed as novel immunotherapeutics with epitope-specific killing potentials, as well as diagnostic reagents (118). Moreover, genetic fusion of *Pseudomonas* exotoxin with TcR-like mAbs amplified the killing tumor cells (121, 122).

These advances, particularly in epitope-specific cancer immunotherapy, seem attractive for the development of TB immunotherapy. Production of mAb and mTcR ligands needs to target some of the empirically identified HLA class I immunodominant epitopes, which have been mapped for a number of Mtb antigens (26, 33, 34, 91, 123, 124). However, a similar approach to MHC class II-presented epitopes could be much more difficult, because their expression is impaired in infected macrophages (125) and because the procedures for producing the corresponding TcR-like ligands are yet underdeveloped. However, some concerns need to be addressed: immunodominance of the currently known MHC class I epitopes may have resulted from peptide presentation by dendritic cells or by cross-presentation, while a TcR-based immunotherapy would need to be targeted against epitopes expressed by infected macrophages. Therefore, it is a prerequisite to confirm the specificity and density of epitope expression on Mtb-infected macrophages. This needs to be ascertained by their capacity to stimulate CD8 T-cell clones or better by direct detection of the levels of epitope expression by antibody staining (126), or by tandem mass spectrometry (127). Notably, the latter technique showed that epitope abundance does not necessarily associate with the immunodominance hierarchy of epitopes.

Since apoptosis of macrophages is known to be the key mechanism for the killing of intracellular mycobacteria (128, 129), conjugation of TcR-specific ligands with apoptosis-inducing agents may amplify the therapeutic effect. Suitable candidate compounds for this purpose, with proven apoptosis-inducing capacity are *Pseudomonas* exotoxin A (130), granzyme B (131, 132), or BH3 peptide (133, 134).

Further synergistic benefit may come from recombinant INFγ treatment, which enhances the surface expression of MHC-bound epitopes and has even alone been therapeutically beneficial in TB patients (135).

## Tregitopes

Tregitopes are epitopes, mostly in the Fc and constant Fab region of IgG, with highly conserved structure between mammalian species. They bind promiscuously to HLA class II molecules and stimulate and expand CD25(+) FoxP3(+) natural regulatory T cells (nTreg) (136). Tregitopes were shown to inhibit CD8 T-cell responses to co-administered antigens, with potentials to prevent or treat autoimmune disease, e.g., Type 1 diabetes or suppress allo-specific responses in mouse models. Co-administration of Tregitopes and auto-antigens reduced diabetes in NOD mice, while the *in vitro* response of T cells from diabetic patients to GAD65 epitopes was found suppressed by Tregitopes (137). Tregitopes might also prevent immune responses against hyper-variable Ig Ids, generated by somatic mutations.

The long-known therapeutic effects of intravenous human gamma globulin therapy (ivG) in autoimmune or allergic diseases, organ transplantation, and graft-versus-host disease have been attributed to the presence of Tregitopes in IgG (136, 138). The proposed mechanism as ivG-induced immunological tolerance has been supported by the increase of Treg cells and IL-10 production after ivG treatment. This concept is relevant to TB, considering the finding that intranasal or intraperitoneal inoculations of human gamma globulin inhibited the BCG viable counts in the lungs of intranasally infected mice (139, 140). Although the authors attributed this effect to the action of specific antibodies, an alternative possible explanation could involve the role of Tregitopes.

Increased Treg numbers in patients with active TB depress the IFNγ-secreting T-cell response to a protective antigen, such as the heparin binding hemagglutinin (141). Mycobacterium-activated human CD8 Treg cells co-express CD39, which is involved in the suppression of CD4 Th1 cell proliferation, lymphocyte activation, and express also LAG-3 and CCL4 (142). Impairing the function of Tregs in mice reduced Mtb infection (143), but antibody inactivation of Tregs, which increased the immune response did not affect the bacterial load after infection (144) and did not influence protection by BCG vaccination (145). In view of these discrepancies about the possible Treg function in TB and the lack of knowledge about their specificity, the possible role of Tregitope recognition deserves further study.

## Conclusion

Epitope specificity of immune responses of Mtb-infected hosts is significant for the immunopathogenesis of TB and its knowledge is mandatory for the development of new approaches toward TB control. HLA-permissive epitopes may have evolved in the tubercle bacilli due to the advantage from immune reactions, which lead to protracted transmission of the infection. Further research needs to expand knowledge on associations between epitope specificity with different effector and regulatory T-cell populations. It is proposed that combining of the biosignature of the T-cell phenotype with epitope specificity might lead to the discovery of protection and disease-associated biomarkers. There are important potentials toward the future development of epitope-defined diagnostics, prophylactic vaccines, and immunotherapies.

## Conflict of Interest Statement

The author declares that the research was conducted in the absence of any commercial or financial relationships that could be construed as a potential conflict of interest.
